# Na-Influenced Bulk and Surface Properties of the So-Called Iota(ι)-Alumina: Spectroscopy and Microscopy Studies

**DOI:** 10.3389/fchem.2021.633877

**Published:** 2021-02-22

**Authors:** Ali Bumajdad, Shamsun Nahar, Mohamed I. Zaki

**Affiliations:** ^1^ Chemistry Department, Faculty of Science, Kuwait University, Safat, Kuwait; ^2^ Chemistry Department, Faculty of Science, Minia University, El-Minia, Egypt

**Keywords:** iota-alumina, bulk characterization, surface characterization, adsorptive interactivity, acid-base catalytic activity, Na-influences

## Abstract

The test alumina (the so-called ι-Al_2_O_3_) was thermally recovered at 1,100°C from chitosan-AlO_x_ hybrid films and found to contain Na and Ca impurity ions inherited from the parent chitosan. Two different modifications of pure alumina, namely, γ- and α-Al_2_O_3_, were adopted as control samples. The test and control aluminas were examined for 1) the bulk elemental constitution by atomic absorption spectroscopy (AAS), 2) the surface chemical composition by X-ray photoelectron spectroscopy (XPS), 3) the bulk phase composition by X-ray powder diffractometry (XRD), *ex-situ* Fourier-transform infrared spectroscopy (IR), and Laser Raman (LRa) spectroscopy, 4) the surface area, topography, and morphology by N_2_ sorptiometry, and atomic force (AFM) and scanning electron microscopy (SEM), 5) the surface adsorptive interactions with pyridine and 2-propanol gas-phase molecules by *in-situ* IR spectroscopy of the adsorbed species, and 6) the surface catalytic interactions with 2-propanol gas-phase molecules by *in-situ* IR spectroscopy of the gas phase. Results obtained could clearly show that the test alumina (ι-Al_2_O_3_) is only hypothetically pure alumina since in reality its bulk structure is majored by mullite-type Na-aluminate (Na_0.67_Al_6_O_9.33_/NaAlO_2_) and minored by Na-β-alumina (Na_1.71_Al_11_O_17_) and β-alumina (NaAl_11_O_17_). Consistently, observed Na-influenced modifications of the surface chemistry, topology, and morphology, as well as adsorptive and catalytic interactions with pyridine and 2-propanol gas-phase molecules, showed significant deviations from those exhibited by the control pure aluminas (γ- and α-Al_2_O_3_).

## Introduction

Iota-alumina (ι-Al_2_O_3_) is, hypothetically, the end-member of the compositional series (Al_2_[Al_2+2*x*_Si_2-2*x*_]O_10-*x*_) of mullite minerals (Schneider et al., 2015; Lenz et al., 2020), where *x* corresponds to the number of oxygen vacancies per unit cell. It is supposed to be composed of pure alumina (i.e., *x* = 1) and assumes a mullite-type orthorhombic crystal structure. It was first disclosed in the literature by Forster (1959), who reported in 1959 that he managed to obtain a silica-free and, hence, ι-Al_2_O_3_ via quenching of cryolite-alumina melts. Very recently, Lenz et al. (2020) have revealed several subsequent reports accounting for the formation of ι-Al_2_O_3_ via various methods; Saalfeld (1962) found sillimanite-like orthorhombic crystals of pure Al_2_O_3_ on Al_2_O_3_/Ni-cermets produced at ca. 1,700°C; Duvigneaud (1974) synthesized alkali-doped aluminas and Perrotta and Young (1974) produced silica-free aluminate crystallized from gels in the (Na,K)_2_O-BaO-Al_2_O_3_ system; Korenko et al. (2008) and Kucharík et al. (2010) obtained ι-Al_2_O_3_ in deeply under-cooled cryolite-alumina melts from rapid solidification process; and Ebadzadeh and Sharifi (2008) reported that they have synthesized ι-alumina phase via sol-gel processing of a mixture of dissolved aluminum nitrate and carboxymethyl cellulose.

According to Lenz et al., (2020), none of these reports communicated structural information. It is worth noting, however, that Fischer et al., (1994) published for ι-Al_2_O_3_ only a hypothetical structure model, and Aryal et al., (2011) simulated this model based on density functional theory (DFT). Moreover, Aryal et al., (2011) described ι-Al_2_O_3_ as being a low-pressure, high-temperature phase of transitional alumina that had been argued to precede γ-Al_2_O_3_ in the processing of alumina. These authors (Aryal et al., 2011) have added that ι-Al_2_O_3_ has higher total energy, more ionic bonding character, and lower density than both γ-Al_2_O_3_ and α-Al_2_O_3_, and it is transformed into η-Al_2_O_3_ on the way to α-Al_2_O_3_.

Motivated by the strong, interdependence of ι, γ, and η polymorphs of alumina, as revealed by Aryal et al., (2011), our laboratories have successfully synthesized chitosan-AlOx hybrid films of different AlOx contents (5, 10, and 20 wt%), whose thermal degradation products (at 500°C) helped the recovery of γ-/η-Al_2_O_3_ (at 800°C) and ι-Al_2_O_3_ (at 1,100°C) (Al Sagheer et al., 2018). The verification of the ι-phase was based on the X-ray diffraction data filed in the JCPDS card no. 12-0539 (ICDD, 2005), as well as those simulated by Aryal et al., (2011). It has been concluded (Al Sagheer et al., 2018) that the higher the purity and the thermal stability of the ι-phase, the lower the AlOx-content (≤10%) of the hybrid film. In contrast, the 1,100°C calcination product of a chitosan-free mixture, or a hybrid film of higher AlOx content (>10%), was predominated by the thermodynamically stable α-Al_2_O_3_ phase (Wefers and Misra, 1987).

Our investigation, however, was stimulated by the following prominent conclusions of the recent work by Lenz et al., (2020): 1) the compounds identified in others works (Perrotta and Young, 1974; Ebadzadeh and Sharifi, 2008; Korenko et al., 2008; Kucharík et al., 2010) as being a mullite-type ι-Al_2_O_3_ are Na-aluminate mullites [Na_0.67_Al_6_O_9.33_], which, based on that the described synthesis routes dwell sodium, 2) the works by Saalfeld (1962) and Duvigneaud (1974) could not provide convincing arguments in support of the existence of ι-Al_2_O_3_, 3) hitherto, there has been no crystal-structure description of the ι-Al_2_O_3_ phase, and the DFT-derived simulated structure (Aryal et al., 2011) is very unlikely to represent a stable compound, 4) the mullite-type Na-aluminate is stable to heating up to 1,000°C, where it commences to transform into a Na-β-aluminate phase [Na_1.66_Al_11_O_17_], and at 1,050°C another β-aluminate phase [NaAl_11_O_17_] emerges, which remain stable up to 1,400°C. Bearing these conclusions in mind, one may envisage the obvious reliance of the formation of the putative ι-Al_2_O_3_ in our earlier investigation (Al Sagheer et al., 2018) to the availability of chitosan in the hybrid film, as implying presence of sodium in the chitosan. The fact that chitosan is a natural polysaccharide that is extractable from marine waste via chitin (Kumirska et al., 2010) may imply the presence of alkali metal ions.

Therefore, the present investigation was designed to recover ι-Al_2_O_3_ from chitosan-AlOx hybrid films as described earlier (Al Sagheer et al., 2018) and search for bulk and surface sodium in the chitosan and the recovered alumina by using atomic absorption (AAS) and X-ray photoelectron (XPS) spectroscopy. If present, Na-influenced modifications of the so-called ι-alumina bulk phase composition, as well as of its surface accessibility, topography, morphology, and acid-base properties (vs. pure γ-Al_2_O_3_ and α-Al_2_O_3_ for control purposes) employing N_2_ sorptiometry, atomic force (AFM) and scanning (SEM) electron microscopy, *ex-situ* Fourier-transform infrared spectroscopy (IR), Laser Raman (LRa) spectroscopy, and *in-situ* IR spectroscopy of pyridine adsorption and 2-propanol catalytic decomposition. Hence, the present investigation aims essentially at the reality of the so-called ι-Al_2_O_3_ via a proper follow-up of Na-impacts on the material bulk and surface properties. To the best of our knowledge, such an approach to this issue has, hitherto, not been encountered in the literature.

## Experimental

### Materials

The so-called iota-alumina (ι-Al_2_O_3_) was obtained by calcination at 1,100°C (for 3 h in a static atmosphere of air) of degradation product at 500°C (for 9 h in air) of 5 wt%-AlOx containing chitosan-AlO_x_ (CA) hybrid films, as carried out previously (Al Sagheer et al., 2018). The CA films were prepared following the recipe reported elsewhere (Al-Sagheer and Merchant, 2011). Accordingly, chitosan (CS), a practical-grade product of Sigma-Aldrich (degree of deacetylation: 75–85%), was dissolved in 2% acetic acid solution (in deionized water) to produce a 2 wt%-CS solution. The resulting solution was stirred for 48 h at room temperature (RT) for homogeneity. Then, the required amount of the CS solution was mixed (in a 50-ml bottle) with the appropriate amount of 98%-pure Al-ethoxide product of Aldrich, followed by 1 h stirring at RT. Subsequently, a stoichiometric amount of an equimolar mixture of ethanol and water was added to the solution, followed by 18-h stirring at RT to carry out the sol-gel processing. The water/Al-ethoxide molar ratio was kept as 1:4. The resulting mixture was, then, cast in Teflon Petri dish, dried at 50°C for 18 h and left under vacuum for 48 h at the same temperature till the formation of flexible CA hybrid films. Pure γ-Al_2_O_3_ and α-Al_2_O_3_ were used for control purposes. The γ-Al_2_O_3_ was the Aluminiumoxid-C of Degussa (Germany) and was used as obtained, whereas the α-Al_2_O_3_ was obtained by calcination at 1,200°C (for 5 h) of the γ-Al_2_O_3_ (Wefers and Misra, 1987)^.^

Spec-pure liquids of pyridine (C_5_H_5_N) and 2-propanol (C_3_H_8_O) were Merck products (Germany). Aliquots of each were kept in Pyrex-glass traps and deaerated, before use, by freeze-pump-thaw cycles at liquid N_2_ temperature (−195°C). 99.999 %-pure oxygen (O_2_) and 99 %-pure nitrogen (N_2_) gases were supplied, respectively, by KOAC (Kuwait) and IGP (Al-Hawamddyia, Egypt).

### Characterization

The metal constitution of material bulk was determined by atomic absorption spectroscopy (AAS) using a Perkin Elmer–Pinnacle 900 F spectrometer (Germany), whereas the metal constitution of material surfaces was determined by X-ray photoelectron spectroscopy (XPS). The XPS spectra were measured using a VB Scientific 200 spectrometer (United Kingdom) equipped with MgKα radiation (1,253 eV) and operated at 23 kV and 13 mA. Binding energies (in eV) were determined concerning the C1s line (284.6 eV) originating from adventitious carbon. The surface atomic percentages of the observed elements were calculated from the relevant peak area(s) with integral subtraction of the background. On the other hand, the phase composition was elucidated by employing X-ray powder diffractometry (XRD), Laser Raman (LRa) spectroscopy, and *ex-situ* Fourier-transform infrared absorption (IR) spectroscopy. XRD powder diffractograms were measured (at RT) over 2θ = 5–80° using a model D500 Siemens diffractometer (Germany) equipped with Ni-filtered CuKα radiation (λ = 0.15406 nm) and operated with 1^o^ diverging and receiving slits at 50 kV and 40 mA and a continuous scan with a step size of 0.014° and a step time of 0.2 s. An online data acquisition and handling system, powered with DIFFRAC Evaluation software (EVA) and ICDD database (ICDD), was used for refinement of the powder diffractograms, background subtraction, and automatic data matching with standard data patterns for crystalline phase composition identification. However, crystallite sizing was determined using Sherrer’s formula (Snyder, 1999). LRa spectra of the samples were measured on a Renishaw Via Raman microscope (United Kingdom) with He-Ne laser (632.8 nm) as the excitation source. *Ex-situ* IR spectra were taken (averaged 20 scans, at 4,000–400 cm^−1^ and the resolution of 4 cm^−1^) from thin disks of KBr lightly loaded (≤1 wt%) with test samples, employing a Genesis II Fourier-transform infrared spectrometer (Mattson/United States). The thermal stability of the chemical integrity of test materials was examined by thermogravimetry (TG). TG and DTG curves were obtained on heating ca. 10 mg portions of test samples up to 800°C at 10°C/min in a dynamic (50 cm^3^/min) atmosphere of air using a model TA-50 Shimadzu thermal analyzer (Japan) equipped with online data acquisition and handling system.

The surface topography, morphology, and specific area were determined by implementing, respectively, atomic force microscopy (AFM), scanning electron microscopy (SEM), and N_2_ sorptiometry (BET-analysis). AFM images were obtained using a model VEECO-Nano scope IV (United States). SEM images were obtained using JEOL JSM- 7001 F scanning electron microscope (Japan) operating at 120 kV. Test samples were sputter-coated with gold using Balzers SCD-050 sputter coater before the SEM examination. Specific surface area (S_BET_/m^2^ g^−1^) was determined by BET-analysis (Brunauer et al., 1938) of N_2_ physisorption data measured (at −195°C) after test sample degassing at 100°C and 10^–5^ Torr (for 12 h), using ASAP 2020 automatic micromeritics sorptiometer (United States).

### Surface Reactivity

The surface adsorption behavior toward pyridine (Py) and 2-propanol (2-PrOH) gas-phase molecules and catalytic activity toward the 2-PrOH gas-phase molecules were examined using *in-situ* IR absorption spectroscopy and a specially designed all-Pyrex glass IR reactor/cell equipped with CaF_2_ windows (Peri and Hannan, 1960). *In-situ* IR spectra were taken (averaged 20 scans, at 4,000–400 cm^−1^ and the resolution of 4 cm^−1^) from adsorbed species and the gas phase of Py or 2-PrOH on self-supporting waters of test samples placed inside the IR-reactor/cell. Before exposure to 3 Torr Py, or 10 Torr 2-PrOH, a self-supporting wafer of the test sample, mounted inside the IR-reactor/cell, was degassed at a chosen temperature (RT-400°C) and 10^–5^ Torr, with (or without) heating in a stream of 50 cm^3^ O_2_/min for 30 min and a subsequent evacuation while being cooled down to RT. Spectra of the adsorbed species, or gas phase molecules, were obtained after the absorption subtraction of the appropriate background spectrum, facilitated by an online data station powered with Win FIRST Lite v1.02 software (Mattison Corp.). A quantitative analysis was applied to the *in-situ* IR gas-phase spectra, to gauge the catalytic activity of the test samples. The IR νOH-absorption peak at 3,644 cm^−1^ of 2-PrOH was the analytical peak chosen. Getting the integrated area (in cm^2^) of this peak and the amount introduced of the alcohol (in Torr), it was possible to determine a calibration constant (in Torr/cm^2^) for the alcohol. This calibration constant was, then, employed to convert the observed peak area after heating at the chosen reaction temperature (RT-300°C, for 5 min) into the amount (in moles) of the unconverted alcohol. As a result it was possible to determine the alcohol conversion% and the catalyst selectivity (S_as_ = dehydrogenation to give acetone, or S_pp_ = dehydration to give propene), implementing the following equations:

$$\%\text{Conversion}\left(\text{per}\;\text{g}‐\text{cat}\right)=\left[\left({\text{P}}_{\text{i}}‐{\text{P}}_{\text{T}}\right)/{\text{P}}_{\text{i}}\right]\times 100,$$ (1)

where P_i_ = initial alcohol pressure (in Torr) and P_T_ = the alcohol pressure after heating at the reaction temperature (T),

$${\text{S}}_{\text{pp}}=\left[{\text{P}}_{\text{pp}}/\left({\text{P}}_{\text{ac}}+{\text{P}}_{\text{pp}}\right)\right]\times 100,$$ (2)

$${\text{S}}_{\text{ac}}=\left[{\text{P}}_{\text{ac}}/\left({\text{P}}_{\text{ac}}+{\text{P}}_{\text{pp}}\right)\right]\times 100,$$ (3)

where P_ac_ and P_pp_ were pressures of the produced acetone and propene, respectively.

## Results and Discussion

### Material Metal Constitution

#### In the Bulk

AAS results set out in Supplementary Table S1 (in online Supplementary Material) reveal that unlike the cases of the control alumina samples (i.e., γ- and α-Al_2_O_3_), which expectedly show the metal constitution to be made solely out of Al ions, the test ι-Al_2_O_3_ shows additionally the presence of Na and Ca ions. Quantitatively, the Na and Ca proportions are found to be equal to 3.0 and 4.2 wt% of the Al content of ι-Al_2_O_3_, respectively. Supplementary Table S1 shows, moreover, that CS contains detectable amounts of Na and Ca ions, which are inherited initially from the extraction steps of CS from chitin (Kumirska et al., 2010). Hence, the presence of Na and Ca ions solely in ι-Al_2_O_3_ is reminiscent to the presence of chitosan (CS) in the parent hybrid film of the recovered oxide. The bulk metal constitution of ι-Al_2_O_3_ is thereby distinct vs. the control γ- and α-Al_2_O_3_ by the inclusion of Na and Ca ions, in addition to the expected Al ions. Accordingly, γ-Al_2_O_3_ will be handled as pure transition alumina that is used, here, to control the influence of Na (and Ca) on properties of the so-called ι-Al_2_O_3_, which has, reportedly (Aryal et al., 2011), been considered as a further modification of pure transition alumina.

#### On the Surface

Given the AAS results above presented, it is important to find out whether the Na and Ca ions observed in the bulk of ι-Al_2_O_3_ are, also, exposed at the material surface. Therefore, the full XPS spectrum shown in Supplementary Figure S1 was obtained. The spectrum, and the analysis results derived therefrom (Supplementary Table S2), revealed that ca. 83 atom % of the surface chemical composition of ι-Al_2_O_3_ is made up of Al^3+^ (31.09%) and oxide (51.53%) ions, whereas the remaining 17 atom % is shared by C (6.93%), Si (2.55%), Ca (2.11%), and Na (2.05%) ions. The bi-disperse nature of the O1s electron binding energy (BE) over the two peak values of 530.6 and 531.6 eV may reveal that 31.83% of the oxygen detected are due to lattice O^2−^ sites, whereas the rest (19.70%) is due to other oxygen-containing surface groups (e.g., OH^−^/H_2_O) (Briggs et al., 1981). On the other hand, the polydisperse nature of the C1s BE over the three peak values of 284.6, 285.6, and 288.9 eV may account for the presence on the surface of various C-containing species (CH_x_, CO, and COx) (Briggs et al., 1981) and that some of the oxygen detected (19.70%) should, also, be associated with the C-O species detected. The high Na1s BE determined (1,072.0 eV) may be related to Na-O-Al interaction species (Briggs et al., 1981), whereas that of Ca2p (347 eV) may account for the presence of Ca-O-C species (Briggs et al., 1981). Supplementary Table S2 shows, moreover, that implementing the total amount of oxygen detected results in a slightly higher O/Al atomic ratio (= 1.65) than that (= 1.5) expected for pure Al_2_O_3_. However, when only the amount of the lattice O^2−^ sites is implemented, the O/Al ratio is rendered closer to unity. These results may, given the suggested presence of Na-O-Al interaction species, account for a Na-influenced deviation from the typical compositional order of Al_2_O_3_, at least in the surface layer.

### Material Thermal Stability

**FIGURE 1:**
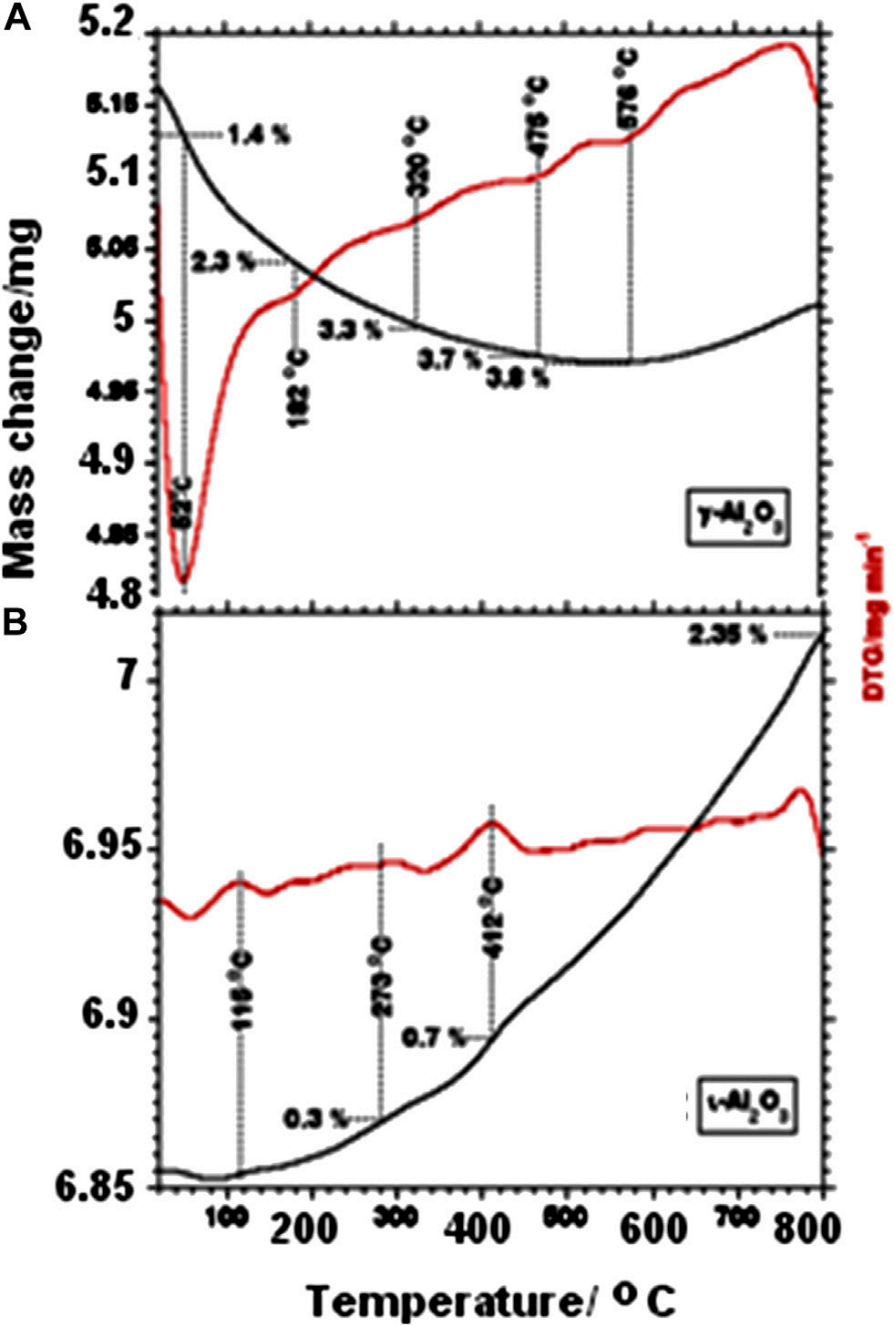
TG and DTG curves obtained for γ-Al_2_O_3_ **(A)** and ι-Al_2_O_3_ **(B)** while being heated at 10°C/min in 50 cm^3^ air/min.

TG and DTG curves obtained for γ- and ι-Al_2_O_3_ while being heated in air are exhibited in Figures 1A,B, respectively. Figure 1A shows γ-Al_2_O_3_ to suffer a total mass loss of ca. 3.8% upon heating up to 576°C, effected through 5 subsequent minor mass loss processes maximized at 52, 182, 320, 475, and 576°C. Literature reports (Zaki and Knözinger, 1987; Knözinger, 2008) may help to ascribe these mass loss events to dehydration processes at the expense of loosely bound water molecules (at ≤100°C) and variously structured surface-OH groups (at > 100°C). Figure 1A shows, moreover, a minute mass gain (ca. 0.03%) at >576°C. Surface dehydroxylation of metal oxides may result in the generation of oxygen vacancies (Knözinger, 2008), whose refilling would attribute this minute mass gain to the uptake of O_2_ and/or peroxide species. In contrast, Figure 1B shows ι-Al_2_O_3_ to suffer a minute mass loss (<1%) at ≤100°C, succeeded by a continuous mass gain of up to 2.35% (at 800°C) resolving broad maxima at 273 and 412°C. It is obvious from these results that the water content of ι-Al_2_O_3_ is much less than γ-Al_2_O_3_, but its tendency toward oxygen uptake is much higher. These behaviors may reveal more hydrophobic and nonstoichiometric and opened bulk structure for ι-Al_2_O_3_ as compared to γ-Al_2_O_3_. This distinctively different thermal behavior of ι-Al_2_O_3_ may logically be ascribed to its contents of Na and Ca ions, which may be envisaged to replace protons of surface-OH groups, and generate oxygen vacancies not only in the bulk but also in the surface layer, which may accommodate the uptake of oxygen at high temperatures.

To emphasize the consequences of the thermal treatment on the surface-OH/H_2_O, *in-situ* IR spectra were taken after a 5 min thermoevacuation of self-supporting wafers of γ- and ι-Al_2_O_3_. The spectra obtained over the νOH frequency region (4,000–3,000 cm^−1^) for γ-Al_2_O_3_ are compared as a function of the thermoevacuation temperature (RT−400°C) in Supplementary Figure S2A. The spectra taken of γ-Al_2_O_3_ are shown to be overwhelmed by the strong and broad absorption of hydrogen-bonded (associated) OH-groups centered on 3,457 cm^−1^ (Zaki and Knözinger, 1987; Knözinger, 2008). Upon increasing the thermoevacuation temperature, this absorption is weakened and, consequently, weaker absorption shoulders for isolated OH-groups are resolved at 3,782, 3,726, 3,666, and 3,573 cm^−1^. According to Knözinger (2008), these absorptions are assignable to a terminal (Al-OH) and bridging (Al..O(H)..Al) surface-OH groups. These spectra may explain the stepwise nature of the mass loss events shown in Figure 1A, since as the dehydration advances, the condensation of remaining OH-groups is rendered more and more difficult (Knözinger, 2008). It is worth noting that an absorption monitored at 1,630 cm^−1^ (not shown) due to δOH vibration of water molecules was almost completely eliminated upon thermoevacuation at 100–200°C. The comparison held in Supplementary Figure S2B reveals without a doubt that the water content of ι-Al_2_O_3_ is much less than γ-Al_2_O_3_ (cf. the difference between the IR absorption strengths of the associated OH-groups on both samples (at RT)). Moreover, the OHs on ι-Al_2_O_3_ are less varied and much less stable to the thermoevacuation (at 400°C) than γ-Al_2_O_3_.

The impact of the water content (water molecules and OH-groups) on the lattice intactness of either γ- and ι-Al_2_O_3_ was revealed via the comparison held, in Supplementary Figure S3, between the lattice edge absorptions before (RT) and after thermoevacuation at 400°C. It is obvious from the figure that the thermoevacuation at 400°C resulted in a larger low-frequency shift in the case of γ-Al_2_O_3_ (= 75 cm^−1^) than ι-Al_2_O_3_ (= 25 cm^−1^). This may reflect the more important role played by water molecules in the coherency of the lattice of γ-Al_2_O_3_ (= 2AlOOH) (Wefers and Misra, 1987) than that of ι-Al_2_O_3_. On the other hand, the much lower content of surface-OH/H_2_O of ι-Al_2_O_3_ may reflect its weaker surface acidity (Knözinger, 2008). It is well known that adsorptive interactions of water molecules with alumina surfaces may occur dissociatively to produce associated and/or isolated surface-Al-OH groups, or nondissociatively to produce coordinated surface-Al…OH_2_ species (Zaki and Knözinger, 1987; Knözinger, 2008). It is obvious from such behavior that the Lewis acidity of the surface Al^3+^ sites plays a key role in defining the affinity of alumina surfaces in adsorptive interactions with water molecules. Accordingly, one may make use of the present results in implying that the Na and/or Ca ions included have caused the observed weakness of the adsorptive interactions of ι-Al_2_O_3_ surfaces with water molecules and, hence, its surface acidity.

### Bulk Phase Composition

**FIGURE 2:**
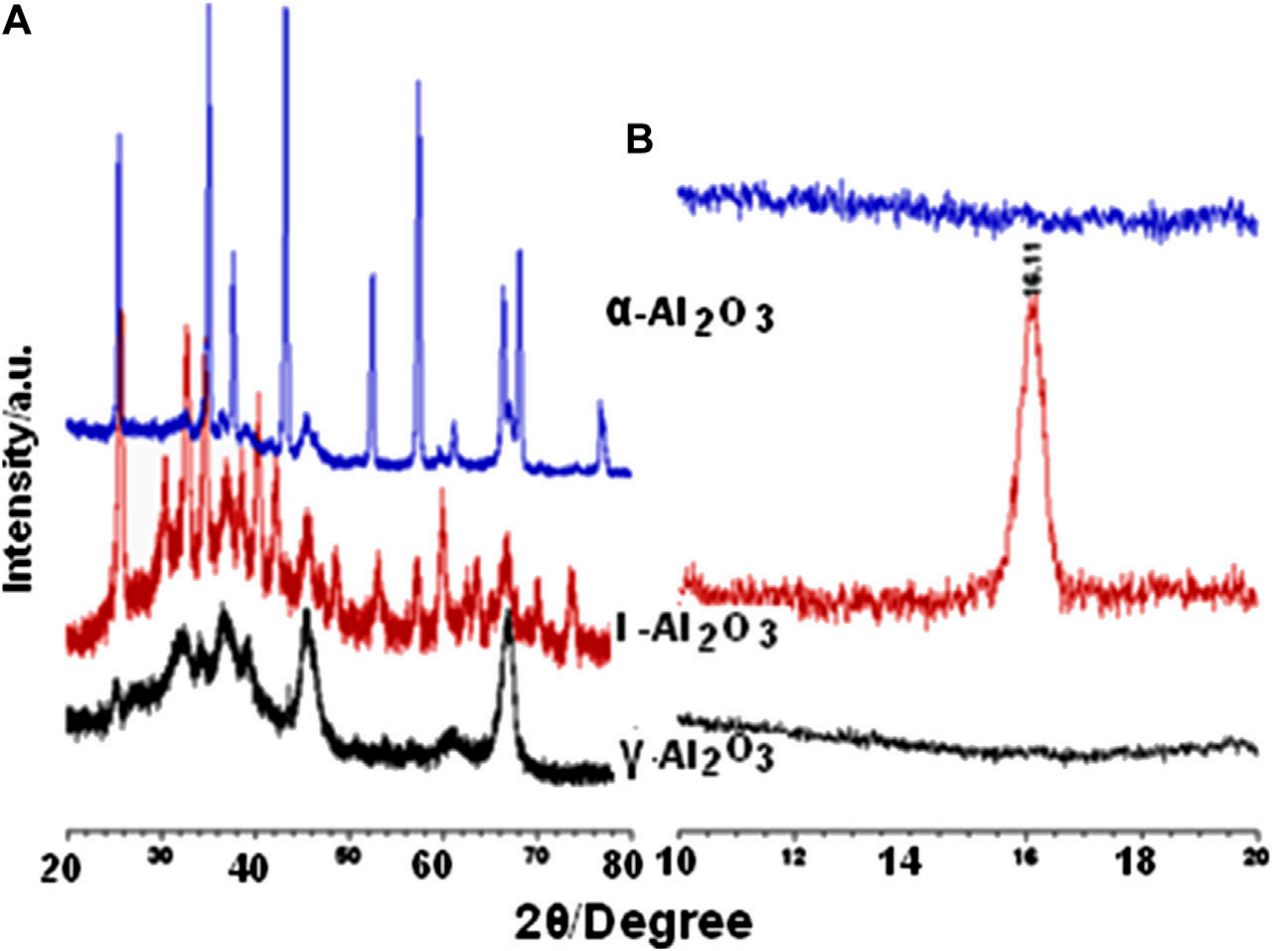
XRD diffractograms obtained for the test and control aluminas at 2θ > 20° **(A)** and <20° **(B)**.

Refined XRD diffractogram obtained for ι-Al_2_O_3_ over the full range scanned of 2θ (5–80°) is exhibited in Supplementary Figure S4. Its original (unrefined) version is compared to the diffractograms obtained (2θ = 10–80°) for the control γ- and α-Al_2_O_3_ in Figure 2. Characteristics of diffraction peaks monitored for ι-Al_2_O_3_ in Supplementary Figure S4 are set out in Supplementary Table S3, alongside those reported for matching crystalline phases. Consulting the ICDD database (ICDD), the diffraction pattern obtained for γ-Al_2_O_3_ (Figure 2) is quite similar to that filed for the cubic-structured pure γ-alumina (Al_2_O_3_) in JCPD card no. 10-0425. On the other hand, the diffraction pattern monitored for α-Al_2_O_3_ (Figure 2) is almost identical to that filed for the rhombohedral-structured pure α-alumina in JCPDS card no. 83–2080. It is worth noting that the diffraction pattern of γ-Al_2_O_3_ has been considered (Wefers and Misra, 1987) representative for the diffraction characteristics of the various polymorphs of the meta-stable transition aluminas (η-, θ- and δ-Al_2_O_3_).

The diffraction pattern obtained for ι-Al_2_O_3_ is shown (Figure 2 and Supplementary Figure S4) to be distinctively different from those obtained for the two control aluminas, particularly over the low angle range (2θ < 20°). The diffraction peaks at 2θ = 7.84 (d = 11.273 Å), 14.42 (d = 6.138 Å), 15.78 (d = 5.612 Å), and 16.78° (d = 5.417 Å) are quite unique for ι-Al_2_O_3_. The observed and reported diffraction peak characteristics compiled in Supplementary Table S3 may help to identify the bulk phase composition as being majored with mullite-type Na-aluminate (Na_0.67_Al_6_O_9.33_) and minored with Na-aluminate (NaAlO_2_). Moreover, the low-angle diffraction peaks (at 2θ = 7.84 and 15.78°) are indicative of the coexistence of minority to trace proportions of Na-β-alumina (Na_1.71_Al_11_O_17_) and β-alumina (NaAl_11_O_17_). This phase composition is almost exactly that reported by Lenz et al., (2020) for ι-Al_2_O_3_ obtained via sol-gel processing of aluminum nitrate and the sodium salt of carboxymethyl cellulose. According to Lenz et al., (2020), the crystal structure of the mullite-type Na-aluminate consists of chains of AlO_6_ octahedra, linked by groups of AlO_4_ tetrahedra, where Na atoms reside in the voids of oxygen vacancies thus generated. Further relevant structural details can be found by Fischer et al., (2001), whereby the pseudotetragonal dimensions (a = b = 7.677 Å, and c = 2.916 Å) expected for alkali aluminate mullite become apparent.

The above-described XRD findings are further summarized in Table 1, which shows, moreover, that the average crystallite size (D = 13 nm) determined for ι-Al_2_O_3_ lies intermediate between the values determined for γ-Al_2_O_3_ (5 nm) and α-Al_2_O_3_ (23 nm). Accordingly, it is the AAS-detected sodium (and not the calcium) retained in ι-Al_2_O_3_ (see Supplementary Table S1) that modified the thermal recovery course of the alumina content of the parent CS-AlO_x_ hybrid film (Al Sagheer et al., 2018) to give rise to the mullite-type Na-aluminate. According to Lenz et al., (2020), the Na-β-alumina and β-alumina are high-temperature conversion products (at ≥ 1,000°C) of the mullite-type Na-aluminate. The fact that the present ι-Al_2_O_3_ was recovered from the parent CS-AlOx >1,000°C (namely, at 1,100°C) may explain the coexistence of minority proportions of the Na-β-alumina and β-alumina in the crystalline domains of the recovered oxide. On the other hand, the presence of these oxygen-vacancy-rich phases may explain the TG-determined mass gain (2.3% at 800°C, Figure 1) due to oxygen uptake by the test ι-Al_2_O_3_. It is worth mentioning that Lenz et al., (2020) came to conclude that it is rather unlikely that a pure ι-Al_2_O_3_ phase exists, thus questioning reports of its formation (Foster, 1959; Perrotta and Young, 1974; Ebadzadeh and Sharifi, 2008; Korenko et al., 2008).

**TABLE 1:** Crystalline phase composition, average crystallite size (D), and specific surface area (S_BET_) of the study aluminas.

Alumina	Phase composition		D/±2 nm	S_BET_/±2 m^2^ g^−1^
	Phase	Abundance^a^		
ι-Al_2_O_3_	Na-aluminate mullite (Na_0.67_Al_6_O_9.33_) +	*j*	13	66
	Na-aluminate (NaAlO_2_) +	*m*		
	Na-β-alumina (Na_1.71_Al_11_O_17_) +	*m*		
	β-alumina (NaAl_11_O_17_)	*t*		
γ-Al_2_O_3_	γ-alumina (2AlOOH = Al_2_O_3_.H_2_O)	*s*	5	77
α-Al_2_O_3_	α-alumina (Al_2_O_3_)	*s*	23	18

^a^j = major, m = minor, t = trace, s = sole

*Ex-situ* IR spectrum taken from ι-Al_2_O_3_ is, also, distinctively different from those exhibited by the control γ- and α-Al_2_O_3_ samples, as shown in Supplementary Figure S5. The same applies to the LRa spectrum taken from the test oxide as compared with that taken from the control γ-Al_2_O_3_ in Supplementary Figure S6. The three IR spectra compared in Supplementary Figure S5 are similar in monitoring absorption bands due to stretching vibrations of hydrogen-bonded OH-groups (at 3,460 cm^−1^), bending vibrations of water molecules (at 1,630 cm^−1^), and lattice vibrations of Al-O bonds (at 820–580 cm^−1^) (Gadsden, 1975). The comparison reveals that the spectrum of ι-Al_2_O_3_ resolves uniquely a strong and sharp absorption at 880 cm^−1^. Its high vibrational energy may account for Al-O bonds involved in Na-O-Al moieties. The comparison further reveals that the νOH and δOH absorptions of ι-Al_2_O_3_ are the weakest, which is intimately related to the least hydrophilic nature concluded for the test oxide from the *in-situ* IR νOH spectra demonstrated in Supplementary Figure S2B. ι-Al_2_O_3_ is shown (Supplementary Figure S5) to be distinct from the control oxides by the higher resolution shown for the lattice vibrations of Al-O bonds, which is most probably related to a more open, less symmetric, and more ionic oxide lattice, most likely due to the formation of Na-O-Al bonds. These IR-revealed distinct lattice properties can well be used to explain the display of LRa peaks (at 1,008, 750, 642, 573, 416, and 376 cm^−1^) in the spectrum obtained for ι-Al_2_O_3_, whereas that obtained for γ-Al_2_O_3_ displays no detectable LRa peaks (Supplementary Figure S6).

### Surface Area, Topography, and Morphology

The BET-determined specific surface area for ι-Al_2_O_3_ is shown (Table 1) to assume an intermediate value (66 m^2^/g) between the values determined for γ-Al_2_O_3_ (77 m^2^/g) and α-Al_2_O_3_ (18 m^2^/g). This trend of variation goes well with the intermediate value (13 nm) assumed by the average crystallite size of ι-Al_2_O_3_ between the values determined for γ- (5 nm) and α-Al_2_O_3_ (23 nm) (Table 1). AFM images compared in Supplementary Figure S7 visualize a more uniform surface topography for ι-Al_2_O_3_ than that visualized for γ-Al_2_O_3_. On the other hand, SEM images displayed in Supplementary Figure S8 reveal particle aggregation for ι-Al_2_O_3_, but only particle coalescence for γ-Al_2_O_3_. AFM and SEM results of ι-Al_2_O_3_ seem to correlate well. The particle sizes are in satisfactory agreement (AFM: ca.151 nm, and SEM: ca.144 nm). In the case of γ-Al_2_O_3_, however, one cannot find any obvious similarity between its AFM and SEM results. Neither the distances (AFM: ca. 500 nm, and SEM ca. 1,200 nm) nor the particle sizes approximate (AFM: 85 nm, and SEM: 175 nm). It is obvious from the present results that the relative high uniformity and order assumed by the surface topography and morphology of ι-Al_2_O_3_ is Na-influenced because such surface features are not assumed by the pure alumina (γ-Al_2_O_3_).

### Surface Reactivity

#### Adsorptive Interactions

##### With Pyridine (Py) Gas-Phase Molecules

**FIGURE 3:**
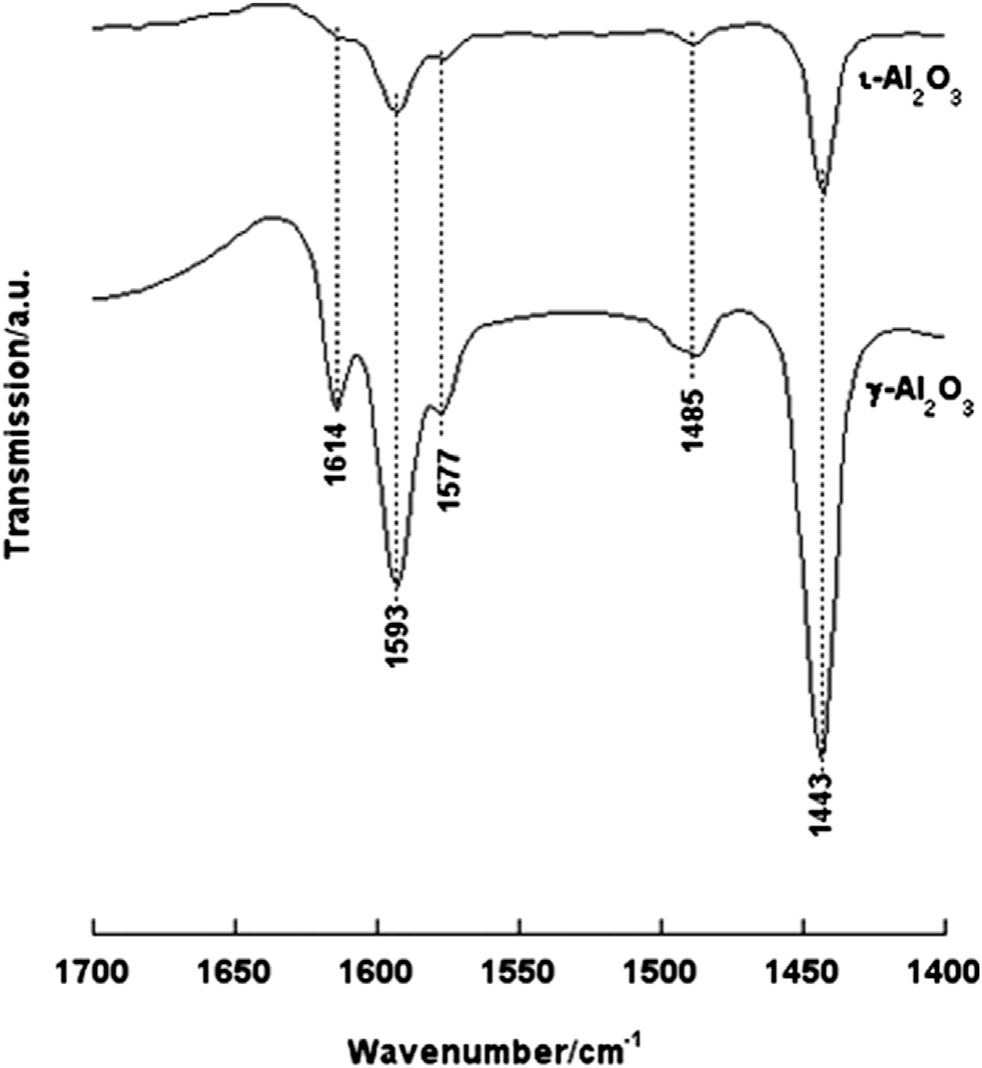
*In-situ* IR νCCN spectra taken from irreversibly adsorbed Py molecules on γ- and ι-Al_2_O_3_ at RT.

*In-situ* IR νCCN spectra taken from irreversibly adsorbed pyridine (Py) molecules on γ- and ι-Al_2_O_3_ at RT are compared in Figure 3. The spectrum taken from Py/γ-Al_2_O_3_ monitors strong absorptions indicating the formation of two types of adsorbed species: (1) hydrogen-bonded Py molecules (HPy at 1,577 and 1,443 cm^−1^) and (2) Lewis acid coordinated Py molecules (LPy at 1,614, 1,593, and 1,485 cm^−1^) (Zaki et al., 2001). The occurrence of the ν_8a_ mode of the νCCN vibration of the LPy species at two different frequency values (1,614 and 1,593 cm^−1^) has been considered indicative of the exposure on the surface of two differently strong Lewis acid sites, namely, coordinatively unsaturated tetrahedral (Al^3+^)_t_ and octahedral (Al^3+^)_o_ aluminum ion sites (Zaki et al., 2001). On the other hand, the formation of HPy species is indicative of the availability on the surface of polarized OH groups capable of hydrogen bonding Py molecules (Zaki et al., 2001). According to Zaki and Knözinger, (1987), these OH-groups are bridging ((Al)_2_OH) and/or multicentered ((Al)_3_OH) surface hydroxyls (cf., the corresponding νOH absorptions at 3,666 and 3,573 cm^−1^, respectively, in Supplementary Figure S2A). The absence of a pair of νCCN absorptions at 1,640–30 and 1,540–1,500 cm^−1^ indicates that none of the exposed surface-OH groups is a proton donor that is capable of protonating Py molecules to form pyridinium ions (PyH^+^); i.e., the surface is void of Brönsted acid sites (Zaki and Knözinger, 1987; Zaki et al., 2001).

The spectrum exhibited for Py/ι-Al_2_O_3_ is also shown (Figure 3) to monitor absorption bands occurring at the same νCCN frequencies of Py/γ-Al_2_O_3_ but of much weaker intensities. These spectral features reveal that ι-Al_2_O_3_ is similar to γ-Al_2_O_3_ in exposing two differently strong Lewis acid sites (i.e., (Al^3+^)_t_ and (Al^3+^)_o_) and hydrogen bonding capable OHs, but seemingly of much less surface population. When the RT-adsorbed Py molecules were exposed to thermoevacuation at 200°C, the spectra obtained showed almost complete desorption of the RT-adsorbed Py molecules on ι-Al_2_O_3_, whereas those adsorbed on γ-Al_2_O_3_ were only partially, but not completely, desorbed. These results indicate that ι-Al_2_O_3_ exposes similarly to γ-Al_2_O_3_ Lewis acid sites associated with coordinatively unsaturated (Al^3+^)_t_ and (Al^3+^)_o_ sites but seemingly of much less acidity strength, and, also similarly, does not expose Brönsted acid sites. Hence, although ι-Al_2_O_3_ has comparably accessible surfaces to γ-Al_2_O_3_ (66 vs.77 m^2^/g), it exposes surface Lewis acid sites of lower population and much less strength than the γ-Al_2_O_3_. This is most likely due to a significant alkalization of the surface which could well be influenced by the XPS-monitored presence of Na^+^ and Ca^2+^ on ι-Al_2_O_3_.

##### With 2-propanol (2-PrOH) gas-phase molecules

*In-situ* IR spectra obtained for irreversibly adsorbed 2-PrOH on γ-Al_2_O_3_ at ≥ RT are compared in Supplementary Figure S9. These are different spectra obtained after the absorption subtraction of corresponding background spectra. Therefore, IR bands of removed or modified species would appear in the negative side of the absorption scale and *vice versa*. Considering the RT-spectrum, the νOH range displays negative absorptions (at 3,783, 3,733, 3,700, and 3,608 cm^−1^) due to isolated surface OH-groups (cf., Supplementary Figure S2) and a broad positive absorption centered on 3,350 cm^−1^ due to associated OH-groups. This reveals that part of the alcohol molecules was molecularly adsorbed via hydrogen-bonding to surface OH-groups. Upon increasing the adsorption temperature, the broad absorption is strongly weakened with the resolution of two maxima at 3,483 and 3,226 cm^−1^. Hence, the H-bonded alcohol molecules are either desorbed or converted, and the persistence of the negative absorptions up to 300°C may mean that they were engaged in the alcohol conversion reactions at the surface. The spectra show, furthermore, νCH absorptions at 2,966–2,875 cm^1^ and δCH/νC-O/νC-C absorptions <1700 cm^−1^. These absorptions indicate dissociative chemisorption of 2-PrOH in the form of surface 2-propoxide species ((CH_3_)_2_CHO^−^) (Hussein et al., 1989; Zaki et al., 1990).

**FIGURE 4:**
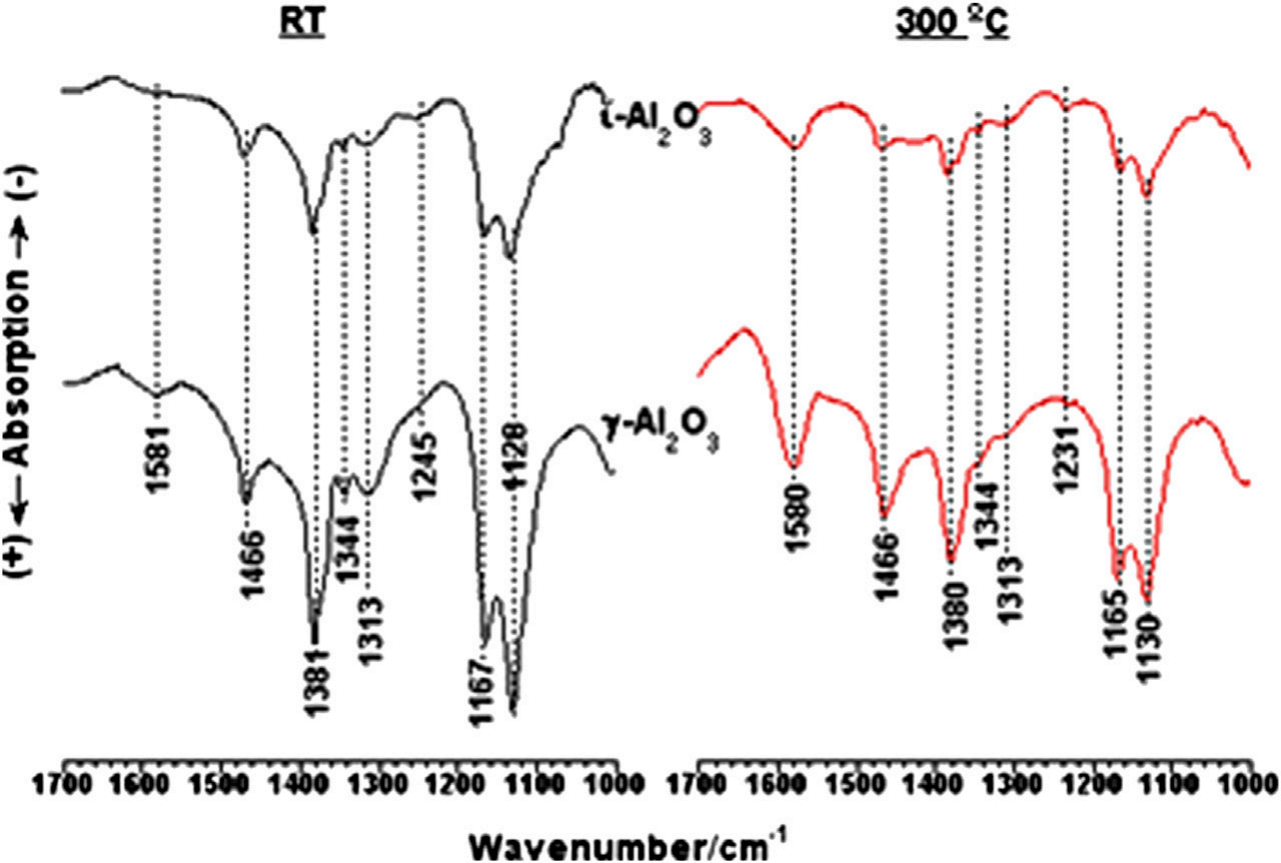
*In-situ* IR spectra obtained for 2-PrOH adsorbed species on ι- and γ-Al_2_O_3_ over the frequency range 1700–1,000 cm^−1^ at the indicated temperatures.

Close-up spectra <1700 cm^−1^ obtained to show the influence of temperature on the IR band structure of 2-propoxide species established on γ- and ι-Al_2_O_3_ are compared in Figure 4. Afar from the weak absorption at 1,581 cm^−1^, all of the absorptions monitored in the RT-spectra at 1,466 (δ(CH_3_)_as_), 1,381 and 1,362 (δ(CH_3_)_s_), 1,344 and 1,313 (δCH), 1,245 (δOH), and the absorptions at 1,167 and 1,128 cm^−1^ (νCO) are assignable to a terminal (νCO at 1,167 cm^−1^) and bridging (νCO at 1,128 cm^−1^) 2-propoxide species (Hussein et al., 1989; Zaki et al., 1990), i.e., (CH_3_)_2_CHO^−^…Al and (CH_3_)_2_CHO^-^ …(Al)_2_. Based on the stronger intensities of the bands of 2-PrOH/γ-Al_2_O_3_, the 2-propoxide species are more populous on γ-Al_2_O_3_ than ι-Al_2_O_3_. The weak absorption at 1,245 cm^−1^ was assigned previously (Hussein et al., 1989; Zaki et al., 1990) to δOH vibrations of surface-coordinated alcohol molecules. Hence, the alcohol molecules are suggested to adsorb molecularly as H-bonded to surface OH/O sites and coordinated to Al^3+^ sites, as well as dissociatively as 2-propoxide species bound to Al^3+^ sites. Upon increasing the temperature up to 300°C, the spectra obtained (Figure 4) show retrogression in the amount of 2-propoxide species, with obvious enhancement in the absorptions at 1,580 and 1,466 cm^−1^, which are indicative of the generation of surface acetate (CH_3_COO^-^) species (Hussein et al., 1989; Zaki et al., 1990). The occurrence of the suggested δOH at the lower frequency of 1,231 cm^−1^ may exclude its relationship to coordinated molecules, thus relating it most likely to minute amounts of carbonate species (Busca and Lorenzelli, 1982). It is obvious from the 300°C spectra that the remaining surface species on ι-Al_2_O_3_ are much less than these remaining on γ-Al_2_O_3_.

So far, it is plausible to consider ι-Al_2_O_3_ as exposing poorly hydroxylated surfaces of weaker overall acidity than the strongly hydroxylated surfaces of γ-Al_2_O_3_. In the literature (Zaki et al., 1990), 2-propoxide formation could occur in two straightforward ways, as in the following reaction equations:

$$2-\text{PrOH}+{\text{L}}^{\text{n}+}\left(\text{s}\right)+{\text{OH}}^{-}\left(\text{s}\right)\to {2-\text{PrO}}^{-}\ldots {\text{L}}^{\text{n}+}\left(\text{s}\right)+{\text{H}}_{2}\text{O}\left(\text{g}\right)$$ (4)

$$2-\text{PrOH}+{\text{L}}^{\text{n}+}\left(\text{s}\right)+{\text{O}}^{2-}\left(\text{s}\right)\to {2-\text{PrO}}^{-}\ldots {\text{L}}^{\text{n}+}\left(\text{s}\right)+{\text{OH}}^{-}\left(\text{s}\right)$$ (5)

where L^n+^ = Lewis acid site, s = surface, and g = gas.

Equation (4) may occur predominantly on hydroxylated surfaces (such as the present γ-Al_2_O_3_), whereas (5) predominates on largely dehydroxylated surfaces (such as ι-Al_2_O_3_). Formation of H-bonded 2-PrOH molecules (2-PrOH…O^2−^(s) and/or 2-Pr(H)O…HO(s)) is followed by low-frequency shifts of νOH vibrations and seems, therefore, to predominate on the largely hydroxylated surfaces of γ-Al_2_O_3_. On the other hand, the formation of coordinated 2-PrOH molecules [2-Pr(H)O…L^n+^(s)] is diagnosed by the emergence of δOH absorption at 1,250–40 cm^−1^ and occurs most likely on dehydroxylated surfaces (such as ι-Al_2_O_3_). Both forms of molecularly adsorbed 2-PrOH (i.e., H-bonded and coordinated species) are thermally less stable than the dissociatvely adsorbed form (i.e., the 2-propoxide species). Therefore, the persistence of the supposed δOH absorption (initially at 1,245 cm^−1^ at RT) and its red-shift to 1,231 cm^−1^ at 300°C may suspect the formation of the coordinated 2-PrOH both at RT and 300°C particularly on ι-Al_2_O_3_. Thus, IR absorption at 1,245–31 cm^−1^ may, alternatively, be attributed to the presence of carbonate species (Busca and Lorenzelli, 1982).

The evident high-temperature formation of surface acetate species (CH_3_COO^−^…L^n+^), more obviously on γ-Al_2_O_3_, could be a consequence of 2-propoxide further reactions (Hussein et al., 1989; Zaki et al., 1990). Its thermal (or oxidative) decomposition may lead to the release of CO_2_ molecules, whose adsorption can lead to the formation of carbonate surface species (${\text{CO}}_{3}^{2-}\ldots {\left({\text{L}}^{\text{n}+}\right)}_{2}$) (Zaki et al., 1990). Since acetate surface species remain observable on γ-Al_2_O_3_ at 300°C, then its considerable disappearance on ι-A_2_O_3_ is more likely to be due, rather, to oxidative conversion into carbonate surface species. Accordingly, one may ascribe this oxidative surface behavior to Na-influenced enhancement of the surface basicity of ι-Al_2_O_3_.

#### Catalytic Activity

*In-situ* IR gas-phase spectra taken from 10 Torr 2-PrOH/γ-Al_2_O_3_ as a function of reaction temperature (RT-300°C, for 5 min) are compared in Supplementary Figure S10. These spectra were obtained after subtraction of the cell background spectrum. The RT-spectrum monitors characteristic absorptions of 2-PrOH gas phase, where the νOH absorption at 3,644 cm^−1^ was the alcohol diagnostic absorption and whose integrated peak area was used to gauge the amount of the unconverted alcohol molecules in the high-temperature spectra. Following heating at 100°C, the spectrum obtained is almost identical to the RT-one, with only 0.5% loss of the initial alcohol concentration. However, upon increasing the reaction temperature to 200°C, a peak at 1738 cm^−1^, assignable to νC=O of acetone molecules (Hussein et al., 1989; Zaki et al., 1990; Zaki et al., 2000), emerged and the alcohol conversion was found to increase up to 21%, whereas a 75% conversion of the alcohol was accomplished at 300°C, with the emergence of absorptions due to propene molecules (at 3,092, 1,652, 1,472 and 1,444 and 920 cm^−1^) (Hussein et al., 1989; Zaki et al., 1990) together with those due to acetone molecules. Moreover, two sharp, but distinct, shoulders emerged at 990 and 900 cm^−1^, which are diagnostic of isobutene molecules (Hussein et al., 1989; Zaki et al., 1990).

Hence, the alcohol decomposition activity of γ-Al_2_O_3_ is shown to be triggered at 200°C via the alcohol dehydrogenation pathway producing acetone molecules (Hussein et al., 1989):

(CH3)2CHO−…Al3+(s)+H−O(s)→(CH3)2CO(g)+H2(g)+O2−(s)+Al3+(s)

The emergence of propene at 300°C is due to the activation of the following alcohol dehydration pathway (Hussein et al., 1989):

(CH3)2CH(H)O…Al3+(s)+O2−(s)→CH3CH=CH2(g)+H−O‐Al3+(s)+H−O(S) (7)

The suggested reaction course (7) is initiated by the formation of coordinated alcohol molecules, which are supposed to form on γ-Al_2_O_3_. Formation of isobutene molecules in the gas phase at 300°C has earlier been attributed to further surface bimolecular reactions of the resulting acetone molecules (Hussein et al., 1989; Zaki et al., 1990; Zaki et al., 2000):

(CH3)2C=O…Al3+(s)+(CH3)2C=O+H−O‐Al3+(s)→(CH3)2C=CH2(g)+CH3COO…−Al3+(s)+H2O (8)

It is obvious that this suggested reaction pathway demands nondissociative adsorption of acetone molecules and explains the observed formation of acetate species on γ-Al_2_O_3_.

**FIGURE 5:**
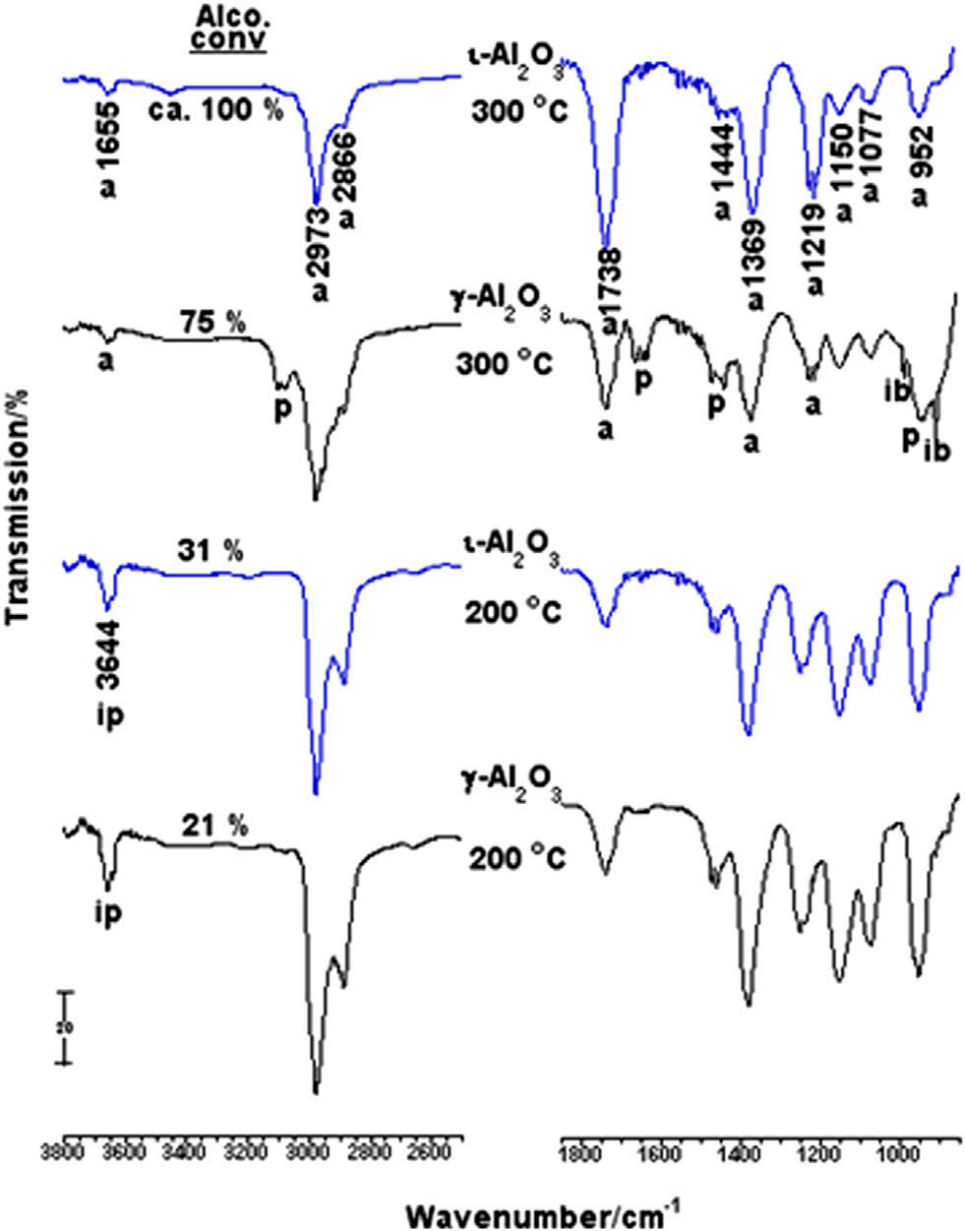
*In-situ* IR gas-phase spectra taken from 2-PrOH/γ- or ι-Al_2_O_3_ following the reaction at 200 and 300°C.

Figure 5 compares spectra obtained for 2-PrOH/γ-Al_2_O_3_ with those obtained for 2-PrOH/ι-Al_2_O_3_ at 200 and 300°C. It is obvious that the alcohol dehydrogenation (to give acetone) on ι-Al_2_O_3_ is similarly activated at 200°C, but with higher alcohol conversion (31%). At 300°C, ι-Al_2_O_3_ is shown to distinctively dehydrogenate almost all of the available 2-PrOH molecules (Conv = ca. 100%) into acetone, without sign of formation of propene molecules. It is rather clear that the Na-influenced surface basicity of ι-Al_2_O_3_ is best manifested in subsiding the known 2-PrOH dehydration selectivity of pure alumina in favor of the dehydrogenation selectivity (Hussein et al., 1989; Zaki et al., 1990). Hence, the Na influenced the transformation of pure alumina (γ-Al_2_O_3_) into a basic catalyst (ι-Al_2_O_3_) capable of 100% decomposition of 2-PrOH into acetone at 300°C. The absence of absorptions due to isobutene in the spectrum obtained at 300°C for 2-PrOH/ι-Al_2_O_3_ may reveal the difficulty of the occurrence of acetone further reactions on ι-Al_2_O_3_ due, most likely, to the Na-influenced weakening of the surface Lewis acidity (cf., Figure 3).

## Conclusion

The above-presented and discussed results may help drawing the following conclusions regarding Na-influenced bulk and surface properties of the so-called ι-Al_2_O_3_.

(1) The presence of adequate proportion of Na ions in the parent material (CS-AlOx) influences the interception of the thermal recovery at 1,100°C (in air) of pure Al_2_O_3_ phase (the so-called ι-Al_2_O_3_) by the formation of Na-aluminate mullite (Na_0.67_Al_6_O_9.33_)/NaAlO_2_ plus minority bulk phases of Na-β-alumina (Na_1.71_Al_11_O_17_) and β-alumina (NaAl_11_O_17_).
(2) The formed Na-aluminate and Na-β-alumina enjoy more open crystalline bulk structures that are rich in oxygen vacancies than pure alumina (e.g., γ-Al_2_O_3_). Therefore, in contrast to pure alumina, they suffer mass gain while being heated in air up to 800°C due most likely to oxygen uptake.
(3) The coexistence of Na on the evolving surfaces influences a more ordered topography and particle aggregation than pure alumina.
(4) Moreover, it turns the rather acidic character of surfaces of pure alumina into basic character, thus rendering its initially dual-functioning alcohol dehydration/dehydrogenation catalytic activity into a solely dehydrogenation-selective activity with optimized alcohol decomposition activity (100% vs. 75% Conversion) at 300°C.
(5) These Na-influenced bulk and surface properties may support the reported XRD-based exclusion of the existence of the so-called pure (Iota-)alumina (Lenz et al., 2020).

## Data Availability

The original contributions presented in the study are included in the article/Supplementary Material; further inquiries can be directed to the corresponding authors.
