# Supplementary material for: Na-Influenced Bulk and Surface Properties of the So-Called Iota(ι)-Alumina: Spectroscopy and Microscopy Studies
Source: Front Chem. 2021 Feb 22;9:633877. doi: 10.3389/fchem.2021.633877 (PMC7937892; doi:10.3389/fchem.2021.633877)
Supplement: Supplementary file 1 [file table1.docx]

*Supplementary Material File*

**Na-influenced Bulk and Surface Properties of the So-called Iota(ι)-Alumina: Spectroscopy and Microscopy Studies**

Ali Bumajdad^1^*, Shamsun Nahar^1^, Mohamed I. Zaki^2^*

^1^ Chemistry Department, Faculty of Science, Kuwait University, P.O. Box: 5969, Safat 13060, Kuwait

^2^ Chemistry Department, Faculty of Science, Minia University, El-Minia 61519, Egypt

*** Correspondence:**Ali Bumajdad
[a.bumajdad@ku.edu.kw](mailto:a.bumajdad@ku.edu.kw)

Mohamed I. Zaki

[mizaki@mu.edu.eg](mailto:mizaki@mu.edu.eg)

**3. Results and Discussion**

**3.1 Material metal constitution**

*3.1.1 In the bulk*

**Table S1.** AAS-determined metal constitution of the test and control aluminas, and chitosan (CS).

| **Solute** | **Al/mg.kg^-1^** | **Na/mg.kg^-1^** | **Ca/mg.kg^-1^** |
| --- | --- | --- | --- |
| **ι-Al_2_O_3_** | 284527.61 | 8720.93 | 12209.30 |
| **γ-Al_2_O_3_** | 306480.61 | - | - |
| **α-Al_2_O_3_** | 291408.56 | - | - |
| **CS** | - | 720.53 | 257.89 |

*3.1.1 On the surface*

**
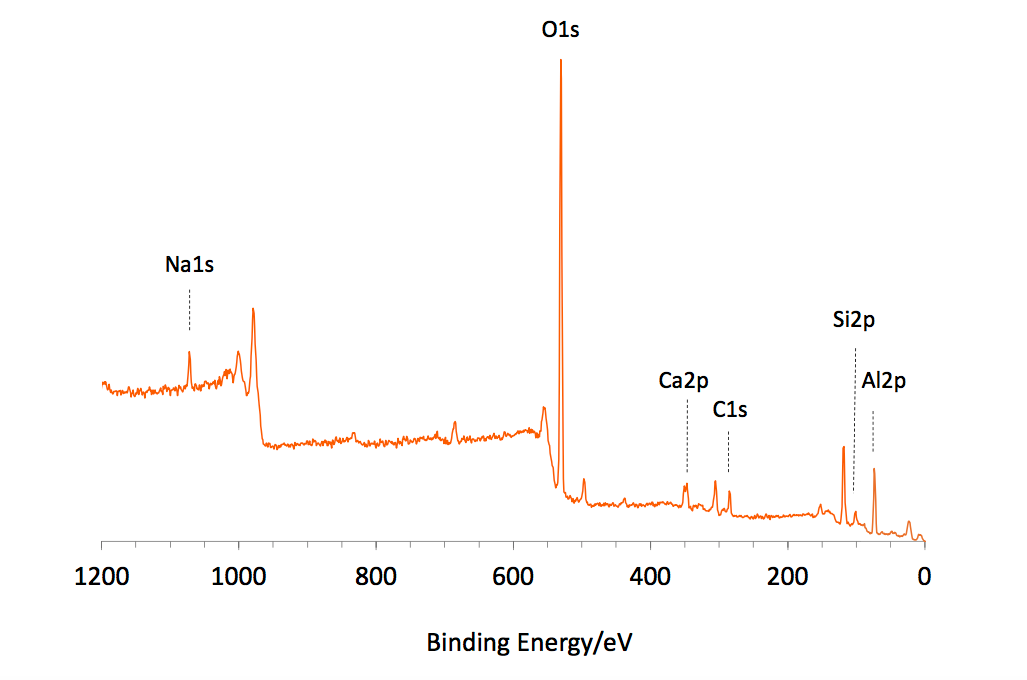
**

**Fig. S1.** The full XPS spectrum obtained for ι-Al_2_O_3_.

**Table S2.** XPS analysis results revealing the surface chemical composition of ι-Al_2_O_3_.

| **Element** | **BE/eV** | **FWHM/eV** | **Atomic %** |
| --- | --- | --- | --- |
| **C1s (A)** | 284.6 | 1.30 | 3.69 |
| **C 1s (B)** | 285.6 | 1.90 | 2.33 |
| **C 1s (C)** | 288.9 | 2.00 | 0.91 |
| **O1s (A)** | 530.6 | 1.50 | 31.83 |
| **O 1s (B)** | 531.6 | 1.74 | 19.70 |
| **Al2p** | 73.7 | 1.64 | 31.09 |
| **Ca2p** | 347.1 | 1.78 | 2.11 |
| **Na1s** | 1072.0 | 2.12 | 2.05 |
| **Si2p** | 101.7 | 1.58 | 2.55 |

**3.2 Material thermal stability**


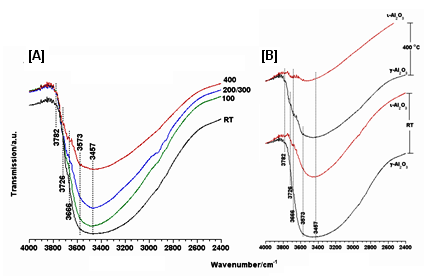


**Fig. S2.** [A] in-situ IR νOH spectra taken of γ-Al_2_O_3_ after 5-min thermo-evacuation at the temperatures indicated, and [Ebadzadeh, #2] a comparison between the spectra obtained after thermo-evacuation at RT (or 400 ^o^C) of γ- and ι-Al_2_O_3_.


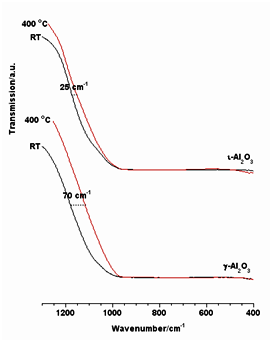


**Fig. S3.** In-situ IR spectra taken over the blackout absorption of the lattice vibrations region before and after thermo-evacuation (at 400 ^o^C) of γ- and ι-Al_2_O_3_.

**3.3 Bulk phase composition**


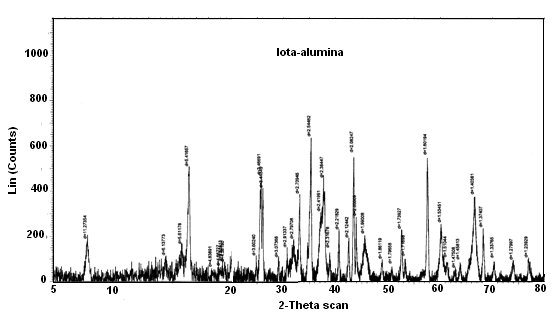


**Fig. S4.** Refined, full XRD diffractogram obtained for ι-Al_2_O_3_.

**Table S3. Observed and reported XRD data for the so-called ι-Al_2_O_3_ and Na-aluminate mullite**

| **Observed** | | | **Reported** | | | | | | | | | |
| --- | --- | --- | --- | --- | --- | --- | --- | --- | --- | --- | --- | --- |
| **So-called ι-Al_2_O_3_** | | | **ι-Al_2_O_3_** | | | | **Na_0.67_Al_6_O_9.33_/NaAlO_2_** | | **NaAl_11_O_17_** | | **Na_1.71_Al_11_O_17_** | |
| (Present work) | | | ^a)^ ([Foster, 1959](#_ENREF_4)) | | ^a)^ ([Ebadzadeh and Sharifi, 2008](#_ENREF_3)) | | ^a)^ ([Lenz et al., 2020](#_ENREF_5)) | | ^a)^ ([79-2288](#_ENREF_1)) | | ^a)^ ([84-0381](#_ENREF_2)) | |
| **2θ**  **/^o^** | **d**  **/Å** | **I/I_o_** | **d**  **/Å** | **I/I_o_** | **d**  **/Å** | **I/I_o_** | **d**  **/Å** | **I/I_o_** | **d**  **/Å** | **I/I_o_^b)^** | **d**  **/Å** | **I/I_o_^b)^** |
| 7.84 | 11.273 | 28 |  |  |  |  |  |  | 11.313 | 100 | 11.180 | 100 |
| 14.42 | 6.138 | 17 |  |  |  |  |  |  |  |  |  |  |
| 15.78 | 5.612 | 24 |  |  |  |  |  |  | 5.656 | 22 | 5.590 | 28 |
| 16.35 | 5.417 | 76 | 5.45 | 100 | 5.41 | 100 | 5.430 | 100 |  |  |  |  |
| 18.32 | 4.839 | 9 |  |  |  |  |  |  | 4.851 | 1 |  |  |
| 19.00 | 4.667 | 10 |  |  |  |  |  |  | 4.743 | 1 | 4.666 | 4 |
| 19.18 | 4.624 | 13 |  |  |  |  |  |  |  |  |  |  |
| 19.36 | 4.581 | 13 |  |  |  |  |  |  | 4.458 | 8 | 4.204 | 9 |
| 20.04 | 4.427 | 26 |  |  |  |  |  |  | 4.080 | 2 | 3.934 | 1 |
| 25.62 | 3.474 | 73 | 3.47 | 100 | 3.42 | 100 | 3.434 | 77 | 3.683 | 1 | 3.727 | 1 |
| 26.01 | 3.422 | 72 |  |  |  |  | 3.435 | 28 |  |  |  |  |
| 29.02 | 3.074 | 19 |  |  |  |  |  |  |  |  |  |  |
| 29.70 | 3.006 | 12 |  |  |  |  |  |  | 2.977 | 1 | 2.805 | 11 |
| 30.63 | 2.917 | 14 | 2.92 | 20 | 2.93 | 20 | 2.917 | 36 |  |  |  |  |
| 31.34 | 2.852 | 15 |  |  |  |  |  |  | 2.828 | 1 | 2.795 | 8 |
| 31.81 | 2.811 | 25 |  |  |  |  |  |  | 2.801 | 6 | 2.760 | 11 |
| 33.03 | 2.710 | 64 | 2.72 | 80 | 2.71 | 80 | 2.715 | 96 | 2.689 | 17 |  |  |
| 34.60 | 2.590 | 27 | 2.59 |  |  |  |  |  | 2.510 | 11 | 2.583 | 16 |
| 35.17 | 2.549 | 100 | 2.59 | 80 | 2.56 | 80 | 2.570 | 76 |  |  | 2.507 | 18 |
| 36.78 | 2.442 | 35 | 2.46 | 5 |  |  | 2.429 | 23 | 2.443 | 2 |  |  |
| 37.14 | 2.419 | 45 |  |  |  |  |  |  | 2.426 | 1 | 2.422 | 8 |
| 37.36 | 2.405 | 51 |  |  |  |  |  |  | 2.411 | 6 | 2.404 | 2 |
| 37.61 | 2.390 | 69 |  |  |  |  |  |  |  |  |  |  |
| 37.78 | 2.379 | 70 | 2.34 | 80 | 2.31 | 80 |  |  | 2.372 | 3 |  |  |
| 38.79 | 2.320 | 17 |  |  |  |  | 2.323 | 38 |  |  |  |  |
| 39.18 | 2.297 | 8 |  |  |  |  |  |  | 2.309 | 1 | 2.333 | 11 |
| 39.39 | 2.285 | 9 |  |  |  |  |  |  | 2.262 | 1 | 2.284 | 2 |
| 40.64 | 2.218 | 24 | 2.24 | 80 | 2.21 | 80 | 2.223 | 52 | 2.229 | 2 | 2.236 | 6 |
| 42.52 | 2.124 | 21 | 2.15 | 40 | 2.15 | 40 | 2.130 | 42 | 2.137 | 7 | 2.166 | 5 |
| 43.42 | 2.082 | 61 |  |  |  |  |  |  |  |  | 2.102 | 1 |
| 44.00 | 2.057 | 31 | 2.06 | 5 |  |  |  |  | 2.040 | 8 |  |  |
| 45.54 | 2.057 | 23 |  |  |  |  |  |  |  |  |  |  |
| 48.89 | 1.990 | 11 | 1.99 | 5 | 1.99 | 5 | 1.920 | 6 | 1.990 | 1 | 1.967 | 23 |
| 50.78 | 1.861 | 8 | 1.88 | 20 | 1.88 | 20 | 1.866 | 19 | 1.841 | 2 | 1.863 | 1 |
| 52.58 | 1.796 | 25 |  |  |  |  |  |  | 1.782 | 1 | 1.794 | 1 |
| 53.31 | 1.739 | 12 | 1.73 | 40 | 1.73 | 40 | 1.720 | 14 | 1.746 | 1 | 1.748 | 1 |
| 57.48 | 1.717 | 60 |  |  |  |  |  |  | 1.699 | 1 | 1.715 | 1 |
| 60.26 | 1.602 | 28 | 1.61 | 20 | 1.59 | 20 | 1.604 | 16 | 1.594 | 5 | 1.610 | 3 |
| 61.33 | 1.534 | 11 | 1.55 | 60 |  |  | 1.538 | 52 | 1.538 | 1 | 1.5314 | 4 |
| 62.96 | 1.475 | 6 | 1.49 | 10 |  |  | 1.480 | 3 | 1.486 | 2 |  |  |
| 63.78 | 1.458 | 10 | 1.46 | 10 |  |  | 1.458 | 32 | 1.440 | 1 | 1.457 | 2 |
| 66.56 | 1.403 | 41 | 1.41 | 5 |  |  | 1.409 | 2 | 1.400 | 11 | 1.403 | 15 |
| 68.19 | 1.374 | 26 |  |  |  |  | 1.359 | 4 | 1.368 | 1 | 1.380 | 12 |
| 70.32 | 1.337 | 11 | 1.35 | 10 |  |  | 1.338 | 17 | 1.336 | 1 | 1.330 | 1 |
| 74.00 | 1.279 | 10 | 1.29 | 60 |  |  | 1.285 | 10 | 1.281 | 1 | 1.273 | 1 |
| 76.86 | 1.239 | 11 |  |  |  |  |  |  | 1.242 | 2 | 1.238 | 1 |
| 79.97 | 1.198 | 6 |  |  |  |  |  |  | 1.211 | 1 | 1.195 | 1 |

**^a)^ Data source reference. ^b)^ I/I_o_ ≥1.**


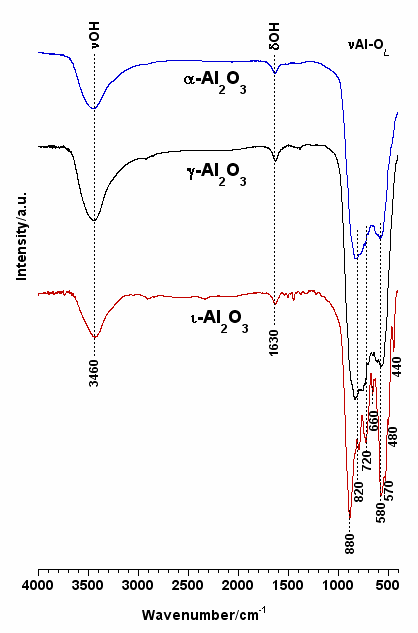


**Fig. S5.** Ex-situ IR spectra obtained for the test and control aluminas.


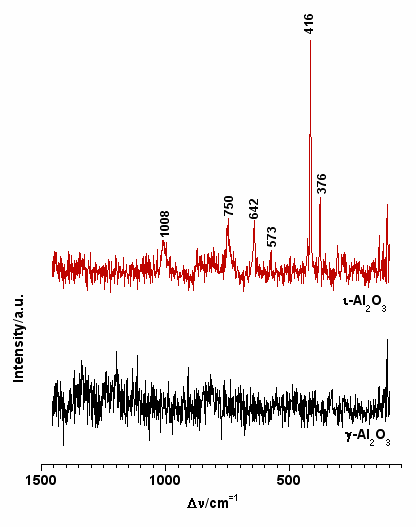


**Fig. S6.** LRa spectra obtained for ι-Al_2_O_3_ and γ-Al_2_O_3_

**3.4 Surface area, topography, and morphology**


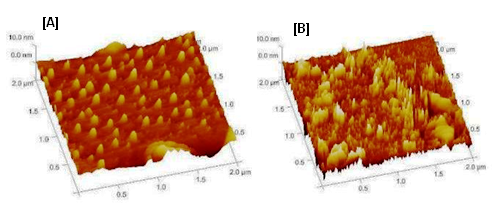


**Fig. S7.** AFM images obtained for ι-Al_2_O_3_ [A] and γ-Al_2_O_3_ [B]


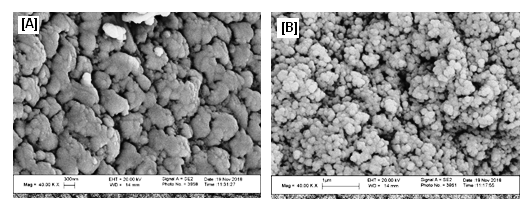


**Fig. S8.** SEM images obtained for ι-Al_2_O_3_ [A] and γ-Al_2_O_3_ [B]

**3.5 Surface reactivity**

*3.5.1 Adsorptive interactions*


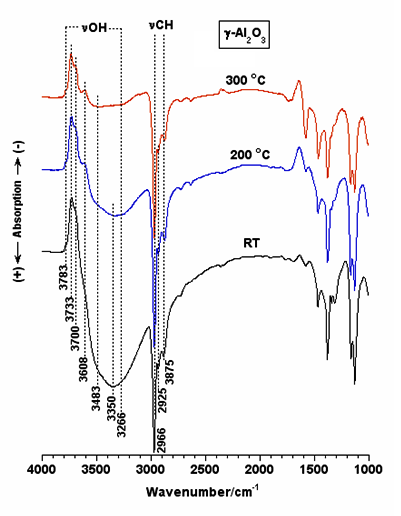


**Fig. S9.** In-situ IR difference spectra obtained for 2-PrOH/γ-Al_2_O_3_ adsorbed species at ≥RT

*3.5.2 Catalytic activity*


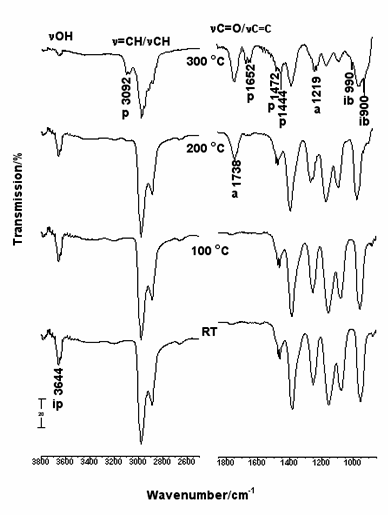


**Fig. S10.** In-situ IR gas phase spectra taken from 10-Torr 2-PrOH/γ-Al_2_O_3_ following 5-min heating at each of the indicated temperatures. [ip = isopropanol; ib = isobutene; a = acetone;

p = prpene].

**References**

79-2288, I.C.F.D.D. "International Center for Diffraction Data", in: *JCPDS 79-2288.* (12 Campus Boulevard, Newtown Square, PA 19073-3273, USA).

84-0381, I.C.F.D.D. "International Center for Diffraction Data", in: *JCPDS Data 84-0381.* (12 Campus Boulevard, Newtown Square, PA 19073-3273, USA).

Ebadzadeh, T., and Sharifi, L. (2008). Synthesis of ι-Al_2_O_3_ from a Mixture of Aluminum Nitrate and Carboxymethyl Cellulose. *Journal of the American Ceramic Society* 91**,** 3408-3409.

Foster, P. A. (1959). The nature of alumina in quenched cryolite-alumina melts. *Journal of The Electrochemical Society* 106**,** 971.

Lenz, S., Schneider, H., and Fischer, R. X. (2020). Mullite-type Na_0.67_Al_6_O_9.33_ and a discussion of iota-alumina. *Journal of the European Ceramic Society* 40**,** 4276-4280.
